# Developing Digital Photomicroscopy

**DOI:** 10.3390/cells11020296

**Published:** 2022-01-16

**Authors:** Kingsley Micklem

**Affiliations:** Nuffield Division of Clinical Laboratory Sciences, Radcliffe Department of Medicine, University of Oxford, Oxford OX3 9DU, UK; kingsley.micklem@ndcls.ox.ac.uk

**Keywords:** histopathology, digital microscopy, fluorescence microscopy

## Abstract

(1) The need for efficient ways of recording and presenting multicolour immunohistochemistry images in a pioneering laboratory developing new techniques motivated a move away from photography to electronic and ultimately digital photomicroscopy. (2) Initially broadcast quality analogue cameras were used in the absence of practical digital cameras. This allowed the development of digital image processing, storage and presentation. (3) As early adopters of digital cameras, their advantages and limitations were recognised in implementation. (4) The adoption of immunofluorescence for multiprobe detection prompted further developments, particularly a critical approach to probe colocalization. (5) Subsequently, whole-slide scanning was implemented, greatly enhancing histology for diagnosis, research and teaching.

## 1. Background

The Mason research group, formally the Leukaemia Research Fund Immunodiagnostics Unit, pioneered the use of monoclonal antibodies in clinical diagnosis. Their key strategy was to screen antibody clones for selection on tissue sections. This had many advantages in that there are a wide variety of cell types on a single microscope slide, and tissue structures are exposed, which are not readily available for binding assay screening. This approach eliminates cross- and pan-reacting clones and selects on utility [1]. This approach was combined with the development of novel, highly specific and sensitive immunostaining methods. Their success required the recording of colour micrographs for presentation and publication. This provided the impetus for the implementation of new methods of photomicroscopy, namely the switch from film to digital.

I first joined David Mason’s research group in 1986 to provide support for the biochemical analysis of the antigens that they were identifying. At the time, there was a backlog in publishing results because of the technical difficulties of providing prints for publication. A dye destruction positive-to-positive photographic process (Cibachrome) was used to produce prints of micrographs captured as transparencies, which was technically exacting and time-consuming. This was particularly critical because the laboratory had pioneered immunostaining, which relied on an accurate colour rendition of chromogens. For example, the dark brown peroxidase 3,3′-diaminobenzidine (DAB) substrate and blue haematoxylin counterstain of their Peroxidase:Antiperoxidase (PAP) technique [2] or the fast red chromogen of their alkaline phosphatase monoclonal anti-alkaline phosphatase (APAAP) [3]. Thus, in addition to the complicated technique of taking a well colour-balanced photomicrograph, there was a further delay in producing an accurate print for publication in a journal.

## 2. Electronic Photomicroscopy

David Mason was particularly keen to see if electronic capture and image processing could speed this up. He knew that I had had some experience in computing, and he and I researched and adopted a video capture system using a broadcast-quality camera (JVC KY-F30) with three monochrome Charge Coupled Device (CCD) detectors. Coupled with a video capture card, we were able to acquire digital micrographs that could be more easily printed with accurate colour. From the beginning, we used Apple Macintosh computers, which were popular in professional media applications and had an excellent range of hardware and software available for dealing with images. This approach was described in detail in a review by Cheong et al. [4]. There were technical problems in this system; for example, the prisms used in splitting the light in the microscope trinocular camera head and the prisms used to separate the colour channels to the three respective monochrome detectors in the camera resulted in a colour balance gradient across the image under certain circumstances. This required a certain amount of correction. In addition, the resolution was limiting, and the tiling of low-power micrographs was adopted to give sufficient resolution.

Output for presentations was achieved using a 35 mm film recorder to transparent film for producing presentations where the images were incorporated into, for example, Microsoft PowerPoint slides. For publication before the advent of electronic submission to journals, the output was to colour negative film for processing it as prints. This required the photoprint house to lock the printer’s automatic exposure and colour balance settings on the first frame of each film, which was a neutral grey. After a certain amount of time, some digital colour cameras became available, and we tested several of the early models. It took some time before they achieved the same quality as the video capture, which was, by its nature, analogue and able to catch colour variations with greater fidelity.

## 3. Implementing Digital Capture

There was, at this time, resistance to adopting digital photomicroscopy because of the limitations of the dynamic range in comparison with 35 mm film and issues of resolution, especially for low-power micrographs, which will be discussed below. Even now, the medico-legal and ethical considerations of the authenticity of published digital images (not just micrographs but all illustrated data), which are susceptible to alteration, remain a challenge. The strategies involved in the adoption of digital imaging in pathology departments were reviewed by Micklem and Sanderson [5].

It is important to realise that most digital colour cameras use a Bayer colour matrix filter over the CCD where there are two green pixels and one each of red and blue. This generally matches the sensitivity of the human eye. A consequence of this is the real colour resolution is much lower for red and blue after interpolation, which has a particular impact, for example, on images of blue haematoxylin and fast red-stained microscope slides.

The issue of camera resolution is critical to good digital photomicroscopy, and initially, few adequate cameras were available. Before very large camera sensors were available, scanning cameras were used, such as the Kontron ProgRes and the Zeiss Axiocam, which sampled the image by moving the sensor with a piezoelectric actuator. These cameras were capable of superb resolution, although their use of a pinhole mask reduced their sensitivity. With the availability of large 2 and 5 MP (megapixel) sensors, scanning cameras have fallen out of use for all but the most specialist applications.

A 40×/NA 1.3 (the highest optical index, the ratio of Numerical Aperture to magnification) apochromat objective image has a resolution of about 10 µm, which when sampled at least twice (the Nyquist sampling theorem), and needs a 4 µm imaging pixel. This requires a high specification camera, for example, a typical 5MP camera producing an image of 2400 × 2000 pixels of size 3.45 µm, which, at the time of writing, will cost you GBP 1800. A histopathologist or a haematologist using lower optical index objectives (e.g., 10×/NA 0.25 or 100×/NA1.4) will perhaps need only the 5.9 µm resolution of a typical 2.3 MP camera.

## 4. Fluorescence Photomicroscopy

By the mid-1990s, the Mason/Gatter lab was expanding its interests to visualising more than one antigen simultaneously on tissue sections and ultimately including gene probes. Naturally, they turned to fluorescent probes, which can be detected independently. A rigorous approach was adopted, in particular, colour camera acquisition being rejected as inadequate.

To achieve full independence or probe detection with minimal crosstalk, the key technique was to use sequential acquisition of two or more fluorochromes. In terms of the camera, this was straightforward in that you could use a monochrome camera with integration (i.e., long exposure) to obtain the required sensitivity. More of a challenge was being able to excite one fluorochrome at a time with no unwanted excitation of the other fluorochromes present. DAPI, which was used as a nuclear counterstain, has significant overlap in emission with fluorescein and rhodamine or Texas red. Similarly, fluorescein and rhodamine overlap in emission spectra. This was particularly important when the colocalization of antigens was being studied [6].

Fluorescent microscopes can be a quipped with individual excitation filters, but in the early days, mechanically changing filters resulted in pixel shifts of each colour plane in the image. We adopted the strategy devised by Dan Pinkel, which was to use an excitation filter wheel and triple dichroic and emission filters. A false colour RGB image was produced by placing red, green and blue (usually DAPI) in their respective colour channels. This produced a colour image roughly familiar to pathologists which aided interpretation.

The purchase of a laser scanning confocal microscope allowed not only higher-resolution imaging but also allowed the imaging in three dimensions, for example, lung tumours where the conformation of the blood vessels can only be visualised in three dimensions [7].

The current immunohistochemical state-of-the-art methods include cyclic tyramide signal amplification, which allows reprobing for multiple antigens sequentially. Confocal laser scanning microscopes fitted with spectral detectors addresses the problem of spectral overlap by spectral unmixing. Such techniques are reviewed by Slack et al. [8]. Current commercially developed systems have been developed to allow at least five antigens and a counterstain to be imaged simultaneously [9]. With such a wealth of data in such images, methods are available to simulate the appearance of a classic brightfield haematoxylin and eosin counterstained immunohistochemical image to provide a more easily interpretable image to pathologists [8].

**Figure 1:**
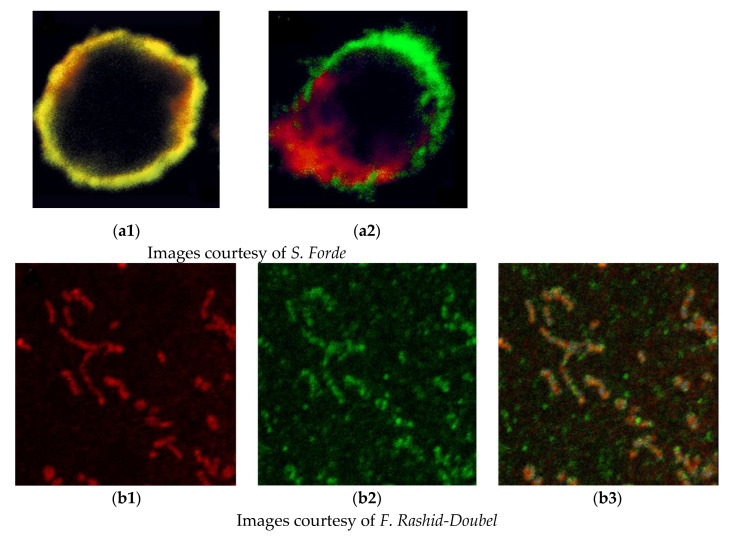
The interpretation of colocalization images. (**a**): Superimposition of immunofluorescence signal does not imply interaction. Stimulation of a stem cell reveals the independent segregation of membrane antigens. (**a1**): Unstimulated stem cell showing strong overlap of red and green signals, which may imply association of antigens. (**a2**): Following stimulation, antigens migrate to opposite poles of the cell, demonstrating they are not associated. (**b**): High-resolution imaging may not produce an overlapped yellow signal even though they are present on the same subcellular organelle. (**b1**): Mitochondria stained with an organelle-specific marker. (**b2**): A new putative candidate antigen for mitochondrial localisation. (**b3**): Combined image showing little yellow signal as the mitochondrial marker is spatially separate from the green-labelled mitochondrial antigen.

One of the common objectives of the simultaneous detection of two or more antigens is to identify colocalization and thus coexpression. Unfortunately, initially, there was a widespread misunderstanding of the commonly used parameters of colocalization, and we sought to provide support and software to allow objective analysis. We recognised that colocalization based on the appearance of a yellow signal caused by the overlap of green and red signals or even correlation coefficients was inadequate and possibly misleading. To quantify the colocalization of fluorescent probes, the objective method of Costes et al. [10] was implemented using specialised software. This involves considering the point spread function of the microscope and the elimination of signal noise by a statistical method. This is still a topic of misunderstanding, and a recent review [11] provides useful guidance. Two examples showing the potential pitfalls in just using yellow signals to identify the association of antigens are given in Figure 1.

**Figure 2:**
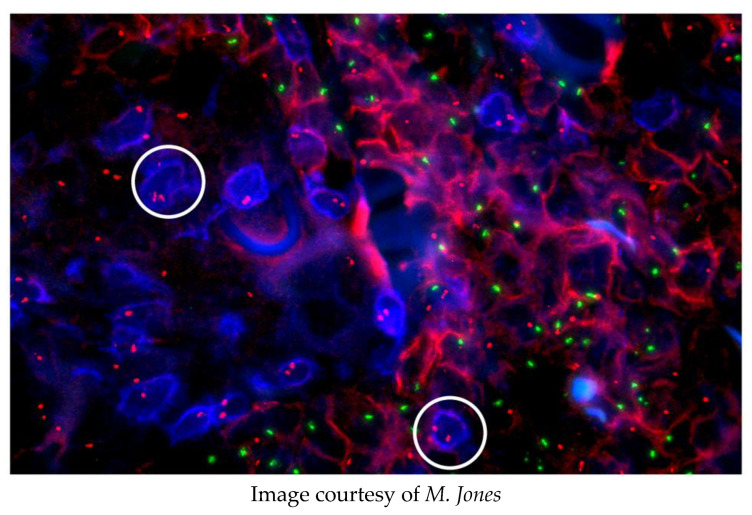
An example of multiprobe imaging.

Ultimately, fluorescence in situ hybridisation (FISH) joined immunofluorescence as a routine technique in the laboratory. This allowed the correlation of genotypic and phenotypic characters of neoplasms, for example, revealing that myelodysplastic syndrome may originate from pluripotent cells able to differentiate into both myeloid and lymphoid cells [12]. An unpublished example of these powerful techniques is given here in Figure 2.

A male patient with myeloid leukaemia was transplanted with his sister’s bone marrow. He subsequently relapsed and was diagnosed with a cutaneous T-cell lymphoma. The origin of this lymphoma was investigated using FISH and immunostaining.

None of the CD3+, T-lymphoma cells (blue, e.g., circled) have a Y chromosome (green spots); the CD54+, myeloid cells (red) have X (red spots) and Y chromosomes. Conclusion: The T-cell lymphoma arose from the sister’s marrow.

A mention must be made of the software which allowed the creation and analysis of digital images. As well as proprietary capture software produced by camera manufacturers, Adobe Photoshop was the preeminent tool. Many camera manufacturers produced capture ‘plug-ins’, allowing the acquisition of images straight into Photoshop. As the program was scriptable, for example, multichannel image capture could be automated. A sophisticated analysis toolkit developed by John C. Russ [13] and marketed by Reindeer Graphics [14], which allowed segmentation and quantitative analysis, was also available as a suite of ‘plug-ins’ for Photoshop. Although we rarely used it, the open-source ImageJ [15] tools were also available.

Subsequently, the group set up a service unit providing imaging and presentation services to the University and Hospital, including wide-field and confocal microscopes and deconvolution software, Huygens from Scientific Volume Imaging [16] for haze removal and 3D imaging.

## 5. Virtual Microscopy

More recent developments include the scanning of tissue microarrays or whole-microscope slides. These ‘virtual slides’ can be viewed remotely using a computer. This has revolutionised histopathology and is being widely implemented in routine clinical laboratories.

An application of ‘virtual slides’ we introduced was the use of annotated teaching slides where hyperlinks in online teaching material were used to specify a particular region of interest and magnification where the student could be guided in self-study. This was extremely popular and greatly facilitated the teaching of histopathology at both an undergraduate and specialist registrar level.

The most recent development in our laboratory is the analysis of scanned tissue sections. Whilst many diagnoses involve a trained pathologist, certain tasks, such as scoring proliferation markers, are amenable to automation. Our work showed that, in general, manual scoring was less susceptible to errors in sample presentation (folded sections, etc.), but that the sheer volume of results produced by automated analysis provided much better statistical confidence in, for example, Ki-67 proliferation indexing. One overnight automated run can replace weeks of work for a busy histopathologist scoring part-time. Automated analysis is still demanding to set up, and scanning issues, such as compression artefacts vs. data size, are difficult to resolve.

## 6. Conclusions

The requirement for accurate full-colour photomicroscopy driven by advances in monoclonal antibody technology followed by the development of new staining methodologies was the impetus for developing these new methods of microscope imaging. Initially, the shortcomings of the available equipment restricted what was possible, but practical solutions were found. The developing technology ultimately removed these shortcomings and eventually allowed new methods to be practicable. Nowadays, the use of virtual slides and the extensive use of digital images in diagnosis, collaboration, teaching and automated analysis have revolutionised histopathology.

It was a privilege to be a part of a pioneering research team at an interesting time during the development of digital imaging, which has transformed clinical microscopy.
